# Non-high-density lipoprotein cholesterol to high-density lipoprotein cholesterol ratio is associated with cardiovascular outcomes after percutaneous coronary intervention in patients with type 2 diabetes

**DOI:** 10.3389/fendo.2026.1844237

**Published:** 2026-05-15

**Authors:** Xi He, Guanlian He, Liqin Qi, Yujie Chen, Zhaorong Lin, Wenjia Chen, Qintao Huang, Libin Liu, Zhaoyang Chen, Ruonan Gao

**Affiliations:** 1 Department of Cardiology, Heart Center of Fujian Province, Fujian Medical University Union Hospital, Fuzhou, China; 2 Department of Endocrinology, Fujian Medical University Union Hospital, Fuzhou, China; 3 Department of Cardiology, Zhongshan Hospital, Fudan University, Shanghai Institute of Cardiovascular Diseases, Shanghai, China; 4 NHC Key Laboratory of Etiological Epidemiology of Chronic Diseases with High Incidence in Fujian-Taiwan Area (Co-construction), Fujian Medical University, Fuzhou, China

**Keywords:** cardiac outcome, myocardial infarction, non-high-density lipoprotein cholesterol to high-density lipoprotein cholesterol ratio, percutaneous coronary intervention, type 2 diabetes

## Abstract

**Background:**

The non-high-density lipoprotein cholesterol to high-density lipoprotein cholesterol ratio (NHHR) is a novel, reliable indicator of dyslipidemia. However, its association with cardiac outcomes in patients with type 2 diabetes mellitus (T2DM) following percutaneous coronary intervention (PCI) remains unclear. We investigated the association of the NHHR with the risks of major adverse cardiovascular events (MACEs) following PCI in adults with T2DM.

**Methods:**

This retrospective cohort study included 1176 adults with T2DM undergoing PCI after new-onset acute myocardial infarction between January 1, 2019, and December 31, 2023. Patients were categorized into NHHR quartiles. The relationship between the NHHR and post-PCI MACE risk was investigated using univariate and multivariate Cox regression analyses. Restricted cubic splines and smooth curve fitting evaluated the nonlinear association between the NHHR and MACE risk; receiver operating characteristic (ROC) curves determined the prognostic significance of the NHHR.

**Results:**

Over 21.0 ± 6.6 months, 246 MACEs occurred. In multivariate analysis, the NHHR was associated with MACE incidence, even after adjustments (vs. group Q1: hazard ratio [95% confidence interval] in groups Q3 and Q4, 4.00 [2.47–6.48] and 6.28 [3.92–10.05], respectively; both *P* < 0.05). The NHHR and MACEs were non-linearly related. Within the threshold of 3.12–5.10, every 1-unit rise in the NHHR elevated the MACE risk 1.4-fold. The area under the ROC curve of the NHHR combined with the Global Registry of Acute Coronary Events risk score was 0.84 for predicting post-PCI MACEs (sensitivity, 71.54%; specificity, 83.86%). The NHHR/MACE association persisted in subgroup analysis. No significant interactive effects were found for age, sex, hypertension, smoking, body mass index, glycated hemoglobin, and medications (P for interaction > 0.05).

**Conclusions:**

Our findings suggested a nonlinear positive association between NHHR and MACE risk in T2DM patients undergoing PCI. Routine NHHR assessment may facilitate early risk identification and tailored care for these patients.

## 1 Introduction

Diabetes mellitus (DM) has reached epidemic levels globally; the prevalence reached 10.5% in 2021 and is predicted to hit 12.5% by 2045 (1). Type 2 DM (T2DM), characterized by insulin resistance and secretion dysfunction, accounts for the most cases. Recently, the global burden of T2DM has rapidly risen, disproportionately affecting younger individuals (2). Prolonged dysglycemia and related metabolic dysfunctions of T2DM lead to various complications involving macro- and microangiopathies.

Myocardial infarction (MI) is the leading cause of mortality in T2DM (3). The risk of first MI within 10 years after diagnosis is greater in patients with T2DM than in those without diabetes. For those patients with a history of MI, recurrence risk exceeds 40% (4). Furthermore, T2DM increases the risk of cardiovascular-related mortality by 1.72-fold compared with the general population (5). Although revascularization largely reduces acute-phase deaths in cases of acute MI (AMI), post-percutaneous coronary intervention (PCI) mortality remains high (6). Therefore, discovering appropriate predictors for the prognosis of AMI in the T2DM population is crucial.

Dyslipidemia is linked to poor clinical outcomes in cases of AMI. Non-high-density lipoprotein cholesterol (non-HDL-C), which comprises low-density lipoprotein cholesterol (LDL-C), very low-density lipoprotein cholesterol, and intermediate-density lipoprotein cholesterol, is a primary target for lowering the risk of coronary artery disease (CAD) (7). In recent years, the ratio of non-HDL-C to high-density lipoprotein cholesterol (NHHR), a composite indicator of lipid metabolism, is considered superior to other lipoproteins in predicting prognosis in patients with metabolic diseases, such as carotid atherosclerosis, as well as adverse cardiovascular events (8). The baseline NHHR was correlated to the incidence of acute coronary syndrome and the advancement of CAD in a previous follow-up study of 7.5 years in patients with CAD (9). Elevated NHHR is also linked to higher MACE rates post-PCI (10), underscoring its potential as an AMI prognostic marker.

Given that dyslipidemia is frequently observed in patients with DM, the utility of NHHR as a predictor of AMI outcomes among patients with T2DM remains unclear. Therefore, we investigated the relationship between NHHR and MACE risk in patients with both T2DM and AMI who underwent PCI. This study will help enhance risk assessment and clinical management for this high-risk population.

## 2 Materials and methods

### 2.1 Study design and population

In this single center, retrospective study, we recruited patients with a history of T2DM or those newly diagnosed with T2DM hospitalized for AMI between January 1, 2019, and December 31, 2023. T2DM was diagnosed in accordance with the World Health Organization guidelines: an elevated level of fasting serum glucose over 7.0 mmol/L, 2-h serum glucose >11.1 mmol/L following an oral glucose tolerance test, or current medication to lower blood glucose. AMI was diagnosed in accordance with the definition by the American College of Cardiology/American Heart Association.

The following exclusive criteria were established: diagnosis of type 1 or another type of diabetes other than T2DM; cancer or other serious diseases; missing data that might influence the statistical results; and loss to follow-up. The study followed the ethical standards of the Declaration of Helsinki and with the approval of the Ethics Committee of Fujian Medical University Union Hospital (**2026KY060**). Given the study’s retrospective design, the requirement for written informed consent was waived.

### 2.2 Demographic and laboratory data collection

Demographic and clinical data of patients, including sex, age at hospital admission, body mass index (BMI), blood pressure, and smoking and alcohol use status, were obtained from electronic medical record systems. The presence of hypertension, duration of T2DM, and current medication use were also recorded. Laboratory parameters, consisting of estimated glomerular filtration rate (eGFR), fasting blood glucose and glycated hemoglobin (HbA1C) levels, and lipid metrics including lipoprotein a, lipoprotein b, triglyceride, total cholesterol, HDL-C, and LDL-C, were assessed using the blood plasma samples collected from fasting patients on admission. The NHHR was calculated as follows:

*NHHR* = (*Total cholesterol* [mmol/L] – *HDL-C* [mmol/L])/*HDL-C* [mmol/L].

Levels of myocardial damage indicators, including troponin I, creatine kinase-MB, high-sensitivity C-reactive protein, and N-terminal pro-B-Type natriuretic peptide, were measured at admission. Left ventricular ejection fraction of AMI patients was assessed via the modified Simpson’s technique as per American Society of Echocardiography guidelines. The Global Registry of Acute Coronary Events (GRACE) risk score was computed as described in a previous study (11).

### 2.3 PCI

PCI was performed after coronary angiography by at least two experienced interventional cardiologists in eligible patients. The number of vessels with stenosis, called diseased vessels, were recorded. The location of the target lesion was categorized into the left main-, left anterior descending-, left circumflex-, and right coronary arteries.

### 2.4 Outcome measurements

The study outcome event was the incidence of MACEs, which included cardiac mortality, nonfatal MI, and target vessel reconstruction. Follow-up was conducted every 6 months from the day of PCI via telephone calls, email correspondence, or outpatient clinic visit, until the occurrence of a MACE. Otherwise, the patients continued to be monitored until the study conclusion.

### 2.5 Statistical analysis

Data were analyzed using R software (Version 4.0.2; https://www.r-project.org) and MedCalc version 11.4 (MedCalc Inc., Ostend, Belgium). Based on the NHHR levels, patients were categorized into four quartiles (Q1–Q4). The Shapiro–Wilk normality test was applied to examine the normality of data distributions. Normally distributed continuous variables are expressed as mean ± standard deviation and were compared via one-way analysis of variance. Non-normally distributed variables are presented as median (interquartile range) and were compared by the Kruskal–Wallis test between groups. Categorical data are presented as numbers (n) and percentages (%) and were compared using the chi-square test. Survival analysis was performed using the Kaplan–Meier method and log-rank test.

Cox regression models tested the correlation between the NHHR and MACE incidence. The initial crude model (Model 1) referred to the univariate Cox regression model. In the multivariate Cox regression analysis, confounders were based on its association with the outcomes of interest, clinically relevant, reported as relevant in the literature, or changes in effect estimates of more than 10% (12). Simultaneously, the variance inflation factor (VIF) of selected variables indicated no significant multicollinearity. Therefore, Model 2 factored in age and sex, whereas Model 3 added controls for hypertension, BMI, smoking status, HbA1C, and drug therapies (angiotensin-converting enzyme inhibitors/angiotensin receptor blockers, β-blockers, statins, metformin, and sodium-dependent glucose transporter 2 inhibitors) in addition to age and sex. Findings from the Cox regression analyses were reported as hazard ratios (HRs) along with their 95% confidence intervals (CIs). Restricted cubic splines and smooth curve fitting analyzed and visualized the non-linear correlation of the NHHR and MACE risk. The inflection point was calculated by the recursion method, and a two-stage Cox proportional risk regression model was used to verify the effect size and CI on both sides of the inflection point.

Subgroup analyses were performed, categorized by sex (female or male), age (< 60 or ≥ 60 years), BMI (<25, ≥25 kg/m2), hypertension (yes or no), smoking (yes or no), levels of HbA1c (< 8.1% or ≥ 8.1%), and use of medications (angiotensin-converting enzyme inhibitors/angiotensin receptor blockers, β-blockers, metformin; yes or no). Likelihood ratio assessments were conducted to evaluate the interaction effects across the subgroups. The prognostic significance of the NHHR was determined by computing the areas under the curves (AUCs) of the receiver operating characteristic (ROC) analysis. Statistical significance was set at *P* < 0.05.

## 3 Results

### 3.1 Baseline data characteristics

**Figure 1:**
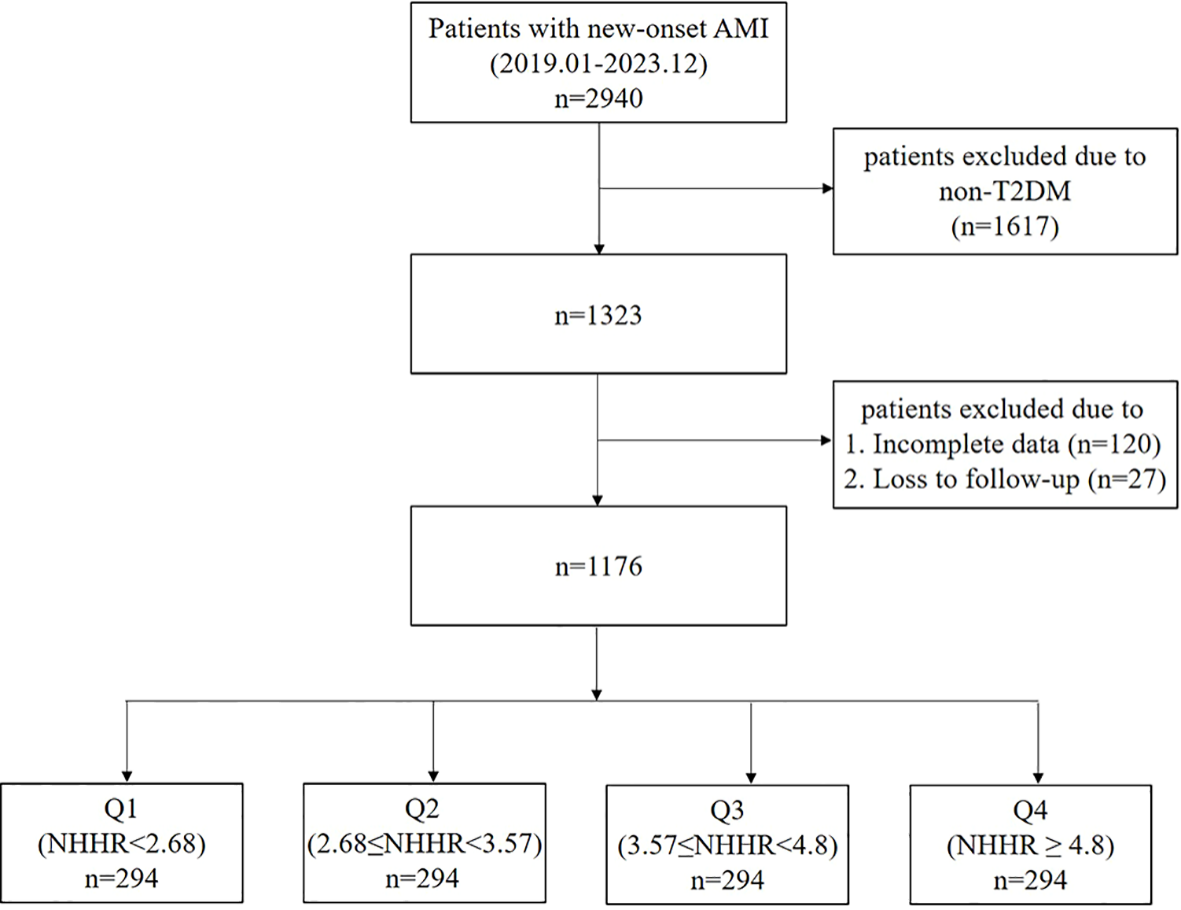
Study flowchart.

In total, 1,176 patients with T2DM were included (**Figure 1**). The average NHHR for the Q1–Q4 groups was 2.10 ± 0.44, 3.11 ± 0.26, 4.14 ± 0.36, and 5.91 ± 1.08, respectively (**Table 1**). Patients in group Q4 were the youngest, had higher BMI, and possessed the greatest serum contents of LDL-C, TC, and lipoprotein b, alongside the lowest contents of HDL-C and lipoprotein a. The Q3 and Q4 groups had higher GRACE scores than the Q1 and Q2 groups.

**Table 1:** Demographic variables and baseline clinical characteristics.

	Q1 (n = 294)	Q2 (n = 294)	Q3 (n = 294)	Q4 (n = 294)	*P*-value
NHHR	2.10 ± 0.44	3.11 ± 0.26	4.14 ± 0.36	5.91 ± 1.08	<0.001
Age (years)	68.63 ± 9.66	66.34 ± 9.89	66.20 ± 10.40	63.35 ± 13.22	<0.001
Sex, n (%)					0.734
Male	222 (75.51%)	210(71.43%)	220 (74.83%)	208 (70.75%)	-
Female	72 (24.49%)	84 (28.57%)	74 (25.17%)	86 (29.25%)	-
BMI (kg/m^2^)	23.78 ± 2.24	24.24 ± 2.66	24.40 ± 2.86	25.23 ± 3.03	<0.001
SBP (mmHg)	129.91 ± 21.59	130.12 ± 21.71	132.10 ± 21.81	129.58 ± 23.57	0.764
DBP (mmHg)	77.46 ± 12.39	77.82 ± 12.77	80.76 ± 14.75	77.86 ± 13.71	0.125
Smoking, n (%)	186 (63.27%)	192 (65.31%)	144 (48.98%)	156 (53.06%)	0.011
Hypertension, n (%)	234 (79.59%)	222 (75.51%)	206 (70.07%)	202 (68.71%)	0.126
Previous MI, n (%)	45 (15.31%)	49 (16.67%)	38 (12.93%)	50 (17.01%)	0.510
Heart failure, n (%)	60 (20.41%)	42 (14.29%)	43 (14.63%)	53 (18.03%)	0.147
Atrial fibrillation, n (%)	53 (18.03%)	53 (18.03%)	48 (16.33%)	66 (22.45%)	0.264
Duration of DM, months	7.06 ± 16.66	12.10 ± 46.04	17.51 ± 69.99	7.25 ± 26.50	0.147
Type of myocardial infarction					0.102
STEMI, n (%)	128 (43.54%)	112 (38.10%)	130 (44.22%)	154 (52.38%)	-
NSTEMI, n (%)	166 (56.46%)	182 (61.90%)	164 (55.78%)	140 (47.62%)	-
Laboratory data					
hsCRP (mg/L)	8.13 (2.63, 23.70)	8.83 (3.34, 19.90)	9.03 (4.59, 28.40)	8.40 (3.26, 19.15)	0.464
TC (mmol/L)	3.49 ± 0.78	4.00 ± 0.84	4.53 ± 0.97	5.24 ± 1.14	<0.001
TG (mmol/L)	1.53 (1.29, 2.30)	1.49 (1.17, 2.03)	1.88 (1.28, 2.33)	1.80 (1.25, 2.59)	<0.001
HDL-C (mmol/L)	1.13 ± 0.26	0.98 ± 0.21	0.88 ± 0.19	0.76 ± 0.20	<0.001
LDL-C (mmol/L)	2.09 ± 0.63	2.62 ± 0.70	3.01 ± 0.88	3.51 ± 1.09	<0.001
apoA1 (g/L)	1.18 ± 0.24	1.10 ± 0.20	1.09 ± 0.22	1.04 ± 0.24	<0.001
apoB1 (g/L)	0.79 ± 0.37	0.89 ± 0.19	1.04 ± 0.22	1.19 ± 0.28	<0.001
FBG (mmol/L)	7.94 (6.47, 11.54)	8.25 (6.47, 11.00)	7.92 (7.14, 10.22)	8.11 (6.20, 11.00)	0.2
HBA1C (%)	8.00 (7.20, 9.15)	7.80 (6.70,9.00)	7.80 (7.00, 8.70)	8.60 (6.80, 10.00)	0.006
eGFR (mL/min/1.73 m^2^)	72.23 ± 31.16	79.82 ± 28.21	77.76 ± 31.47	83.42 ± 36.19	0.024
TnI (ng/mL)	4.18 (0.32, 9.70)	3.37 (0.42, 9.30)	1.19 (0.30, 7.40)	1.61 (0.47, 13.70)	0.335
CK-MB (IU/L)	23.30 (15.80, 82.70)	24.50 (16.55, 70.60)	24.70 (16.55, 66.40)	26.80 (16.25, 130.00)	0.37
LVEF (%)	51.07 ± 11.33	52.84 ± 11.00	54.38 ± 11.76	53.33 ± 9.84	0.074
NT-proBNP (pg/mL)	921.00 (294.00, 2546.00)	1849.00 (383.00, 1995.00)	840.00 (321.00, 2880.00)	690.00 (196.00, 2608.00)	0.192
Medications, n (%)					
DAPT	278 (94.56%)	290 (98.64%)	290 (98.64%)	286 (97.28%)	0.104
Statin	283 (96.26%)	291 (98.98%)	291 (98.98%)	288 (97.96%)	0.055
ACEI/ARB	206(70.07%)	196 (66.67%)	188 (63.95%)	198 (67.35%)	0.738
β-blocker	196 (66.67%)	200 (68.03%)	244 (82.99%)	272 (92.52%)	<0.001
Insulin	64 (21.77%)	74 (25.17%)	54 (18.37%)	56 (19.05%)	0.469
Metformin	90 (30.61%)	68 (23.13%)	84 (28.57%)	64 (21.77%)	0.248
Insulin secretagogues	114 (38.78%)	112 (38.10%)	138 (46.94%)	124 (42.18%)	0.396
DPP-4 inhibitor	16 (5.44%)	24 (8.16%)	50 (17.01%)	54 (18.37%)	<0.001
SGLT-2i	0 (0.00%)	0 (0.00%)	8 (2.72%)	0 (0.00%)	0.007
GRACE risk score	132.22 ± 22.74	133.00 ± 28.71	144.80 ± 27.86	144.18 ± 39.17	<0.001
Hospital stay, days	10.00 (7.00, 13.00)	10.00 (7.00, 13.00)	9.00 (7.00, 12.00)	10.00 (7.00, 12.00)	0.152
Hospitalization costs, CNY	48761.50 (40616.00, 81368.00)	50069.42 (42161.40, 70047.00)	44993.34 (37259.83, 63905.00)	50652.00 (42851.01, 75614.00)	0.098
Number of diseased vessels, (%)					0.043
1-vessel disease	84 (28.57%)	52 (17.69%)	96 (32.65%)	60 (20.41%)	-
2-vessel disease	106 (36.05%)	120 (40.82%)	114 (38.78%)	120 (40.82%)	-
3-vessel disease	104 (35.37%)	122 (41.50%)	84 (28.57%)	114 (38.78%)	-
Location of target lesion, n (%)					0.257
LM	20 (6.80%)	10 (3.40%)	14 (4.76%)	20 (6.80%)	-
LAD	140 (47.62%)	130 (44.22%)	102 (34.69%)	140 (47.62%)	-
LCX	50 (17.01%)	50 (17.01%)	56 (19.05%)	40 (13.61%)	-
RCA	84 (28.57%)	104 (35.37%)	122 (41.50%)	94 (31.97%)	-

ACEI, angiotensin-converting enzyme inhibitors; ARB, angiotensin receptor blockers; apoA1, apolipoprotein A1; apoB1, apolipoprotein B1; BMI, body mass index; CNY, China yuan; CK-MB, creatine kinase-MB; DAPT, dual antiplatelet therapy; DBP, diastolic blood pressure; DM, diabetes mellitus; DPP-4, dipeptidyl peptidase-4; eGFR, estimated glomerular filtration rate; hsCRP, high-sensitivity C-reactive protein; HbA1C, glycated hemoglobin; HDL-C, high-density lipoprotein cholesterol; LAD, left anterior descending artery; LCX, left circumflex artery; LDL-C, low-density lipoprotein cholesterol; LM, left main artery; LVEF, left ventricular ejection fraction; MI, myocardial infarction; NHHR, non-high-density lipoprotein cholesterol to high-density lipoprotein cholesterol ratio; NSTEMI, non-ST-segment elevation myocardial infarction; NT-proBNP, N-terminal pro-B-type natriuretic peptide; RCA, right coronary artery; SBP, systolic blood pressure; SGLT2i, sodium-dependent glucose transporter 2 inhibitor; STEMI, ST-segment elevation myocardial infarction; TC, total cholesterol; TnI, troponin I.

### 3.2 MACE incidence and survival analysis

**Figure 2:**
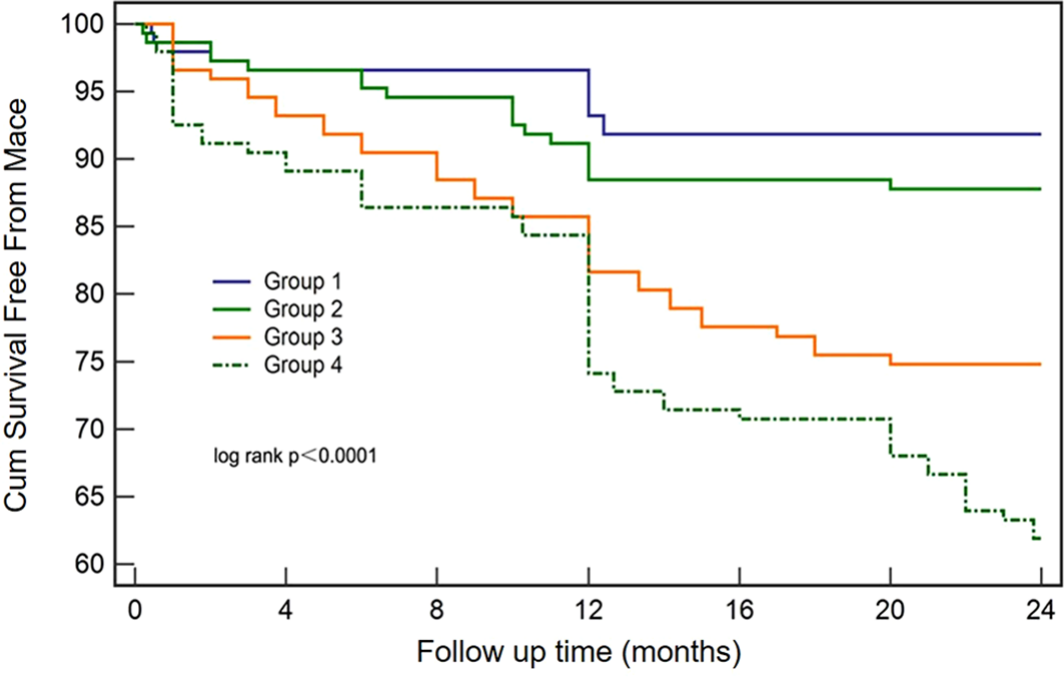
Kaplan–Meier survival analysis of MACEs according to NHHR quartiles. Patients with type 2 diabetes mellitus who underwent percutaneous coronary intervention were categorized by NHHR quartiles: Group 1 (NHHR < 2.68), Group 2 (2.68 ≤ NHHR < 3.57), Group 3 (3.57 ≤ NHHR < 4.8), and Group 4 (NHHR ≥ 4.8). MACE, major adverse cardiac events; NHHR, non-high-density lipoprotein cholesterol to high-density lipoprotein cholesterol ratio.

MACE incidence during the mean follow-up of 21.0 ± 6.6 months were 8.16%, 12.24%, 25.17%, and 38.10% for groups Q1–Q4, respectively (**Table 2**). A higher NHHR significantly correlated with increased post-PCI MACE risk. Specifically, patients in the Q4 group had the highest occurrence rate of nonfatal MI, target vessel reconstruction, and cardiac-related death in the follow-up, compared with those in the other groups. The Kaplan–Meier analysis (**Figure 2**) indicated that the survival rate decreased with the increase in the NHHR, with the Q4 group experiencing the shortest cumulative survival during follow-up.

**Table 2:** Incidence of MACEs during follow-up in patients with T2DM who underwent PCI.

Event	Q1 (NHHR < 2.68)	Q2 (2.68 ≤ NHHR < 3.57)	Q3 (3.57 ≤ NHHR < 4.8)	Q4 (NHHR ≥ 4.8)	*P*-value
MACEs	24 (8.16%)	36 (12.24%)	74 (25.17%)	112 (38.10%)	<0.001
Nonfatal MI	4 (1.36%)	8 (2.72%)	20 (6.80%)	28 (9.52%)	<0.001
TVR	14 (4.76%)	18 (6.12%)	30 (10.20%)	60 (20.41%)	<0.001
Cardiac death	6 (2.04%)	38 (2.72%)	20 (6.80%)	24 (8.16%)	<0.001

MACEs, major adverse cardiac events; MI, myocardial infarction; TVR, target vessel revascularization.

### 3.3 Association of the NHHR with MACEs in patients with T2DM following PCI

Univariate Cox regression analysis (Model 1, **Table 3**) showed that a higher NHHR was correlated with increased MACE risk (HR [95% CI] in Q3 and Q4 vs. Q1 as reference were 3.38 [2.13–5.35] and 5.33 [3.43–8.29], respectively; both *P* < 0.05). Multivariable analysis showed similar trends. In the fully adjusted model (Model 3), MACE risks were 4.00-fold (adjusted HR 4.00, 95% CI 2.47–6.48), and 6.28-fold higher (adjusted HR 6.28, 95% CI 3.92–10.05) in the Q3 and Q4 groups, respectively, than in the Q1 group.

**Table 3:** Association between NHHR and incidence of MACEs in patients with T2DM who underwent PCI.

NHHR	Incidence of MACEs	Model 1 (crude model)		Model 2		Model 3	
(quartiles)	(%)	HR (95% CI)	*P*-value	HR (95% CI)	*P*-value	HR (95% CI)	*P*-value
Q1	8.16	1 (reference)		1 (reference)		1 (reference)	
Q2	12.24	1.54 (0.92–2.59)	0.099	1.59 (0.95–2.68)	0.077	1.68 (0.99–2.84)	0.055
Q3	25.17	3.38 (2.13–5.35)	<0.0001	3.45 (2.17–5.47)	<0.0001	4.00 (2.47–6.48)	<0.0001
Q4	38.10	5.33 (3.43–8.29)	<0.0001	5.63 (3.60–8.81)	<0.0001	6.28 (3.92–10.05)	<0.0001
*P* for trend	-	-	<0.001	-	<0.001	-	<0.001

Model 1 (crude model): no covariables were adjusted; Model 2: adjusted for age and sex; Model 3: adjusted for age, sex, body mass index, smoking, hypertension, glycated hemoglobin, and use of statins, beta-blocker, angiotensin-converting enzyme inhibitors/angiotensin receptor blockers, metformin, and sodium-dependent glucose transporter 2 inhibitors. HR, hazard ratio; CI, confidence interval; MACEs, major adverse cardiac events.

**Figure 3:**
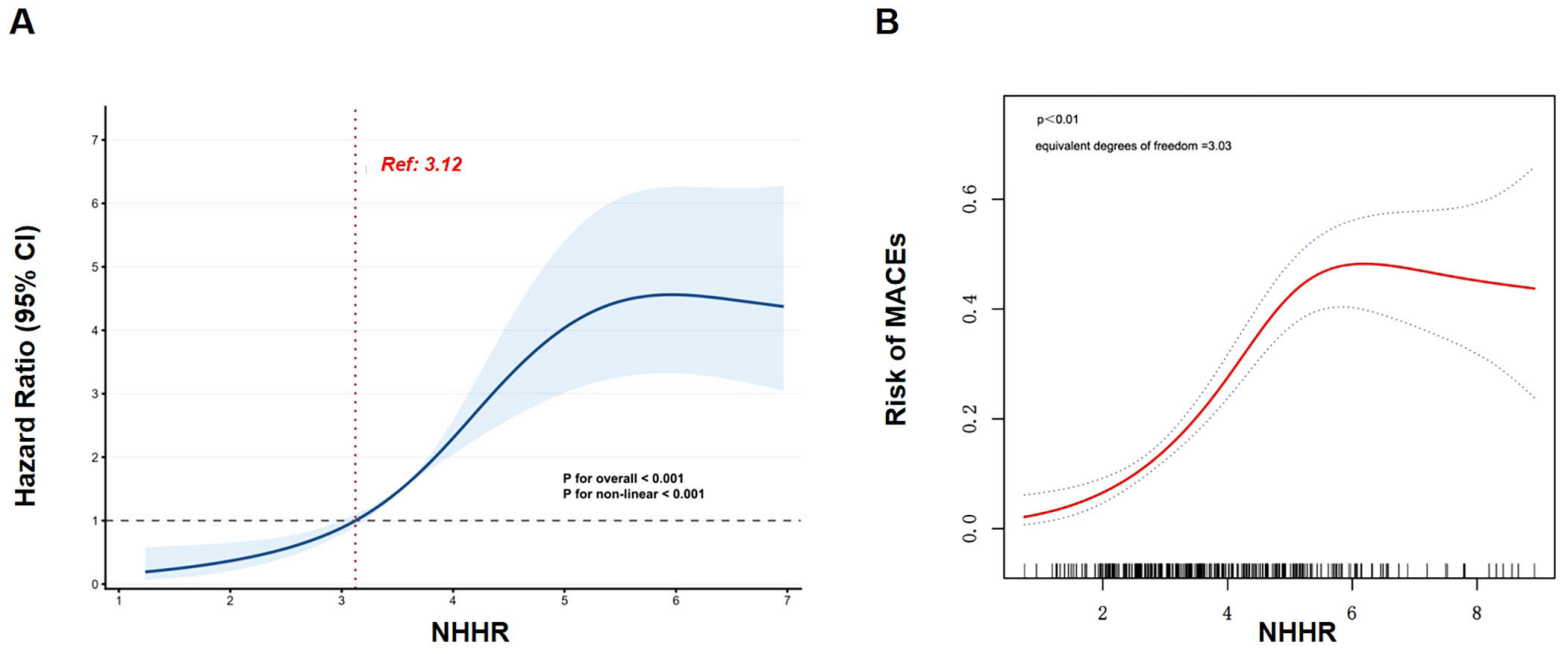
Relationship between the NHHR and risk of MACEs. **(A)** Restricted cubic spline regression analysis demonstrated a nonlinear association between the NHHR and the incidence of subsequent MACEs in patients with type 2 diabetes mellitus who underwent percutaneous coronary intervention. The shaded area represents the 95% confidence interval. The horizontal dashed line demonstrates a hazard ratio of 1.0, and the line of vertical dotted indicates the reference value of NHHR (3.12). **(B)** Smooth curve fitting analysis of NHHR used to estimate MACE risk. Hazard ratios shown are adjusted for Model 3, which consists of age, sex, body mass index, smoking, hypertension, glycated hemoglobin, and use of statins, beta-blockers, angiotensin-converting enzyme inhibitors/angiotensin receptor blockers, metformin, or sodium-dependent glucose transporter 2 inhibitors. MACE, major adverse cardiac events; NHHR, non-high-density lipoprotein cholesterol/high-density lipoprotein cholesterol ratio.

In restricted cubic splines analysis, a nonlinear relationship was found between the NHHR and MACE occurrence in the fully adjusted model (*P*_nonlinear_ < 0.001) (**Figure 3A**), which was further confirmed by smooth curve fitting analysis (**Figure 3B**). The recursive method identified an inflection point for MACE at an NHHR of 5.10. For NHHR values between 3.12 and 5.10, each 1-unit increase elevated the following MACE risk by 1.4-fold (95% CI: 2.0 to 2.8, *P* < 0.05) (**Supplementary Table 1**).

### 3.4 Association of the NHHR with MACEs by subgroups

**Figure 4:**
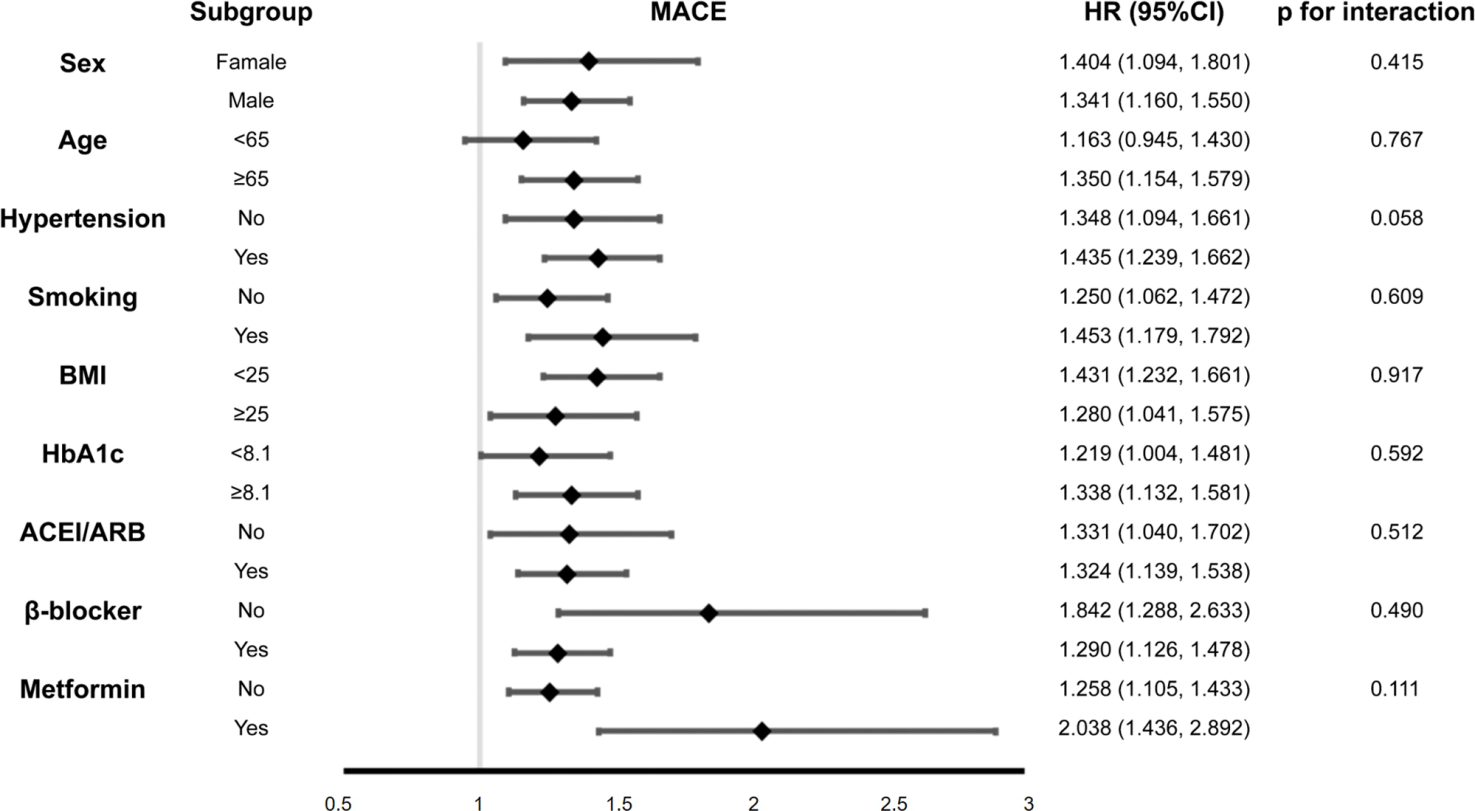
Subgroup and interaction analyses of the association between the NHHR and MACEs. The population was stratified by sex, age (<65 years and ≥65 years), hypertension, smoking, BMI (<25 kg/m^2^; ≥25 kg/m^2^), HbA1C (<8.1% and ≥8.1%), and use of ACEI/ARB, β-blockers, and metformin. ACEI/ARB, angiotensin-converting enzyme inhibitors/angiotensin receptor blockers; BMI, body mass index; HbA1C, glycated hemoglobin; MACEs, major adverse cardiovascular events; NHHR, non-high-density lipoprotein cholesterol/high-density lipoprotein cholesterol ratio.

Subgroup analysis, stratifying patients based on age, sex, history of hypertension, smoking, BMI levels, HbA1C levels, and medications (angiotensin-converting enzyme inhibitors/angiotensin receptor blockers, β-blockers, metformin), demonstrated a persistent association between the NHHR and MACE incidence (**Figure 4**). Interaction tests revealed no significant interactions for all of the abovementioned variables (*P*_interaction_ > 0.05), demonstrating that these confounders did not influence the correlation between the NHHR and post-PCI MACE risks.

### 3.5 Prediction performance of the combined NHHR and GRACE risk score for MACEs

**Figure 5:**
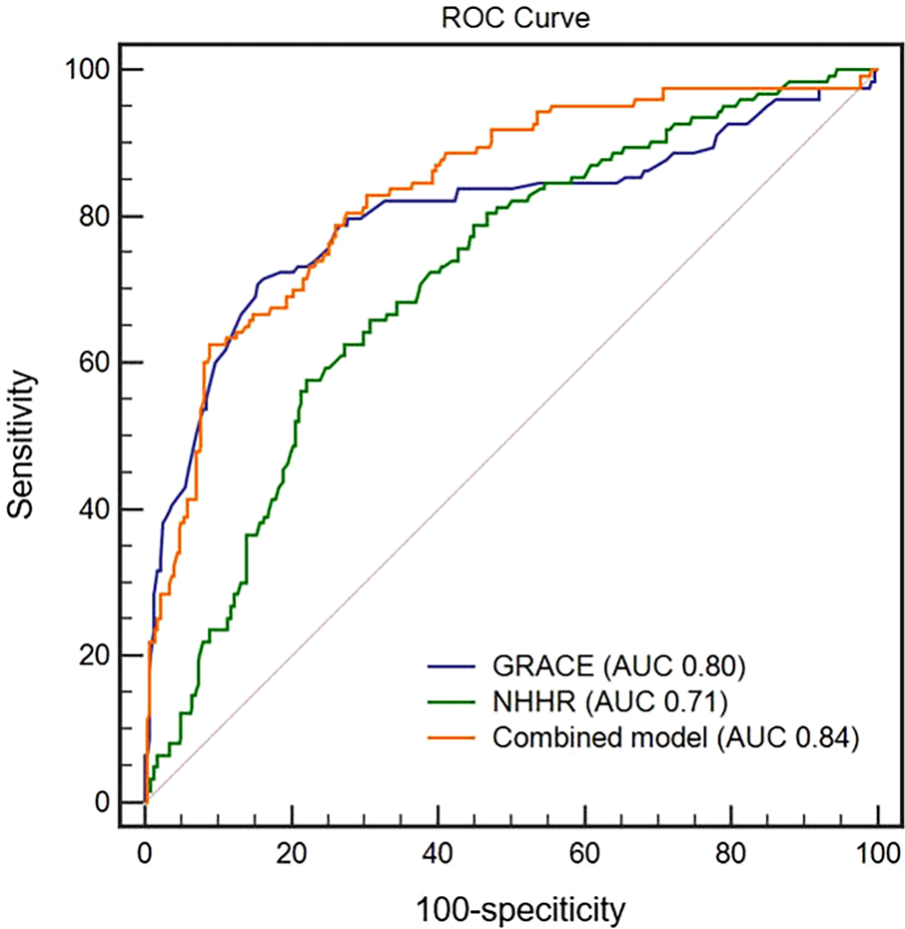
ROC curves of the NHHR, the GRACE risk score, and the combination for predicting MACEs. GRACE, Global Registry of Acute Coronary Event; MACEs, major adverse cardiac events; NHHR, non-high-density lipoprotein cholesterol/high-density lipoprotein cholesterol ratio; ROC, receiver operating characteristic.

In the ROC curve analysis, the AUC for NHHR and the GRACE risk score alone was 0.71 (95% CI: 0.64–0.75) and 0.80 (95% CI: 0.78–0.83), respectively, all *P* < 0.01 (**Figure 5**); on combining these two variables, the AUC value rose to 0.84 (95% CI: 0.81–0.86, *P* < 0.01), indicating a stronger predictive performance. The combined predictors yielded good sensitivity (71.54%) and specificity (83.86%). These results underscore the potential value of a combined NHHR and GRACE risk score in predicting MACE risk in T2DM patients post-PCI.

## 4 Discussion

This retrospective cohort study highlights the relationship between the NHHR and MACE incidence in patients with T2DM following PCI for AMI. We observed a significant association between the baseline NHHR and cardiovascular outcomes of AMI in this population. Subgroup analysis further verified the positive correlation between the NHHR and subsequent MACEs, which was not influenced by age, sex, hypertension, smoking, BMI, HbA1C, and medication use. ROC analysis showed an AUC of 0.71 for the NHHR, which increases to 0.84 when combined with the GRACE risk score. These findings indicate the significance of the NHHR as a prognostic marker for poor cardiovascular outcomes in patients with T2DM who undergo PCI for AMI.

Although PCI is the primary emergency intervention for coronary heart disease, nearly 20% of patients who have undergone successful revascularization still experience subsequent adverse coronary events, such as coronary restenosis or bleeding (13, 14). This proportion is even higher in cases of T2DM, where cardiovascular complications account for 70%**–**80% mortality (15). Furthermore, the risk of adverse cardiovascular events post-PCI is notably greater for adults with DM than for those without (16). Given the rising prevalence of T2DM, identifying clinical prognostic indicators is crucial for managing patients with T2DM following PCI.

Disordered lipid metabolism is a major contributor to cardiovascular complications in the T2DM population. Lowering LDL-C by 1.0 mmol/L lowers the risk of MACE by 21% in patients with T2DM (17). Moreover, there is a correlation between coronary heart disease and other cholesterol fractions such as very low-density lipoprotein cholesterol and intermediate-density lipoprotein cholesterol (18). As a novel comprehensive biomarker, the NHHR represents the lipid composition, outperforming the traditional lipid parameters in predicting various metabolic diseases (19–21). A positive correlation has been identified between the NHHR and cardiovascular disease among individuals aged 45 years and older, and elevated baseline and cumulative NHHR levels increase the cardiovascular disease risk by approximately 27%–41% (22). In populations with diabetes or prediabetes, the NHHR was positively correlated with the risks of all-cause and cardiovascular mortality (23). Moreover, in patients with T2DM, each 1-unit elevation of the NHHR increased the MACE risk by 12% (24). These studies lend indirect support to the conclusions of our research.

While the NHHR correlates with cardiovascular mortality, its impact in patients with T2DM who have undergone PCI after AMI remains unclear. Liu et al. reported that the NHHR exceeding the threshold of 3.119 was positive correlated with MACE risk in patients with CAD following PCI (10). Similarly, we observed that patients with T2DM exhibiting a higher NHHR at baseline, when hospitalized, had a higher incidence of MACE after PCI. Cox regression analysis further validated this strong positive relationship, even after adjustment for age, sex, BMI, smoking, biochemical parameters, a history of hypertension, and medications. This finding suggests that higher baseline NHHR levels predict poor cardiac outcomes after PCI in patients with T2DM.

Several possible mechanisms might explain these findings. Elevated NHHR may result from the increased concentrations of non-HDL-C or reduced HDL-C concentrations. Non-HDL-C, such as LDL-C, has pro-inflammatory effects on cardiovascular diseases (25, 26), and its accumulation in the inner wall of blood vessels promotes the formation of atherosclerotic lesions, thereby inducing coronary artery spasm or even vascular occlusion (27). Furthermore, reduced HDL-C levels, which are involved in reverse LDL-C transport and inhibition of LDL-C oxidation, may aggravate the progression of vascular lesions, facilitate thrombus formation, and reduce plaque stability (28–30). An elevated NHHR has been associated with weakened formation of collateral coronary circulation (31), suggesting impaired myocardial compensatory capacity, which increases the susceptibility to MACEs in patients with T2DM post-PCI.

The restricted cubic splines analysis revealed an S-shaped correlation between the NHHR and MACE incidence. When the NHHR value fell in the range of 3.12 and 5.10, every 1-unit rise significantly elevated MACE risk by 1.4-fold. This finding suggested that during the ascending stage of the S-shaped curves (3.12 < NHHR < 5.10), significant cardiovascular benefit would be obtained by lowering the NHHR value. However, no significant effect was observed when the NHHR value exceeded 5.10, implying that limited benefit is obtained from lowering the NHHR at extremely high levels. This suggests that stricter lifestyle management and health guidance aimed at reducing NHHR are necessary to lower the risk of subsequent MACEs. Notably, the HR for MACE risk approximated 1 when the NHHR value was approximately 3.12, suggesting a potential threshold for clinical intervention to reduce the MACE risk in the T2DM population undergoing PCI. These findings align with those of the study by Liu et al, which also revealed a non-linear S-like relationship between the NHHR and cardiovascular outcomes in patients with T2DM based on the ACCORD cohort (24). However, a previous study found a U-shaped association between baseline NHHR and MACE incidence (10), in which the patients with diabetes account for only nearly 20% of the total study population. The discrepancy might arise from the different population and disease constitution among the studies. In fact, a higher intensity of statin therapy is recommended for patients with diabetes to obtain a much lower LDL-C level than for those without (32, 33). It was reported that lower the LDL-C level is, greater the clinical benefit would be attained in the patients (34). We also confirmed in the present study that a lower NHHR value, which might be due to lower LDL-C levels, was associated with decreased MACE risk in patients with T2DM after PCI, suggesting an S-shape but not U-shape association between NHHR and MACE incidence.

Our subgroup analyses, classified by age, sex, smoking status, BMI, HbA1C, history of hypertension, and history of medication, demonstrated a consistent association between NHHR and post-PCI MACE risk. In predicting the cardiovascular outcomes in patients with T2DM post-PCI, the AUC from ROC curve analysis showed that integrating the NHHR and GRACE risk score, which is a reliable marker of the severity of acute coronary syndrome, performed better than GRACE risk score alone. To the best of our knowledge, only few studies have compared the predictive value of the NHHR in terms of adverse cardiovascular outcomes with that of the GRACE risk score in this population. These findings underscore the prognostic application of the NHHR for patients with T2DM following PCI for AMI. Given its cost-efficiency, ease of ascertainment, and predictive effectiveness, routine NHHR monitoring can help identify risk early and establish targeted management strategies for patients with T2DM after PCI.

Some limitations in this study should be acknowledged. First, this single-center cohort study included only Chinese participants with T2DM who underwent PCI after AMI, implying that the applicability of these findings to broader or more diverse populations may be limited. Second, the retrospective study design prevented the assessment of dynamic NHHR fluctuations during follow-up. Third, owing to the relatively short follow-up period, the association of the NHHR with long-term cardiovascular outcomes could not be evaluated. Future multi-center studies in diverse populations are warranted to explore the superiority of baseline or dynamic changes in the NHHR in terms of predicting long-term MACE risk. Despite these limitations, the present study also has some noteworthy strengths. To the best of our knowledge, no previous study has investigated the relationship between the NHHR and subsequent MACE risk in the T2DM population following PCI. The adjustment for covariates, as well as subgroup analyses, further strengthened our finding of an association between the NHHR and cardiovascular outcomes in this population. Moreover, our ROC-based analysis highlights the clinical utility of combining the NHHR with the GRACE risk score to predict MACE and to facilitate risk stratification and management for patients with T2DM post-PCI.

In conclusion, our results indicated that an elevated NHHR is associated with an increased risk of MACE following PCI in the T2DM population, even after accounting for multiple potential confounders. This relationship between the NHHR and MACE risk is nonlinear. The GRACE risk score combined with the NHHR can greatly improve the prediction of cardiovascular outcomes in these patients. Based on our findings, routine NHHR assessment may aid early detection of risk and the implementation of personalized medical plans in patients with T2DM undergoing PCI for AMI.

## Data Availability

The raw data supporting the conclusions of this article will be made available by the authors, without undue reservation.

## Glossary

NHHR: non-high-density lipoprotein cholesterol to high-density lipoprotein cholesterol ratio
DM: diabetes mellitus
T2DM: type 2 diabetes mellitus
PCI: percutaneous coronary intervention
MACEs: major adverse cardiovascular events
MI: myocardial infarction
ROC: receiver operating characteristic
AMI: acute MI
non-HDL-C: Non-high-density lipoprotein cholesterol
LDL-C: low-density lipoprotein cholesterol
CAD: coronary artery disease
BMI: body mass index
HbA1C: glycated hemoglobin.
