# Supplementary material for: Non-high-density lipoprotein cholesterol to high-density lipoprotein cholesterol ratio is associated with cardiovascular outcomes after percutaneous coronary intervention in patients with type 2 diabetes
Source: Front Endocrinol (Lausanne). 2026 May 15;17:1844237. doi: 10.3389/fendo.2026.1844237 (PMC13218897; doi:10.3389/fendo.2026.1844237)
Supplement: Supplementary file 1 [file Table1.docx]

**Supplementary Table S1.** Analyses of the threshold effect of the non-high-density lipoprotein cholesterol/high-density lipoprotein cholesterol ratio on major adverse cardiac events.

| **Outcomes** | **Linear-regression model** | **Inflection point (K)** | **＜ K, effect 1** | **＞ K, effect 2** | **p-value for LRT** |  |
| --- | --- | --- | --- | --- | --- | --- |
| MACEs | 1.6 (1.4, 1.8) | 5.1 | 2.4 (2.0, 2.8) | 0.9 (0.7, 1.1) | < 0.001 |  |
|  | p < 0.001 |  | p < 0.001 | 0.404 |  |  |

The effect analysis was adjusted for Model 3, which included age, sex, body mass index, smoking, hypertension, glycated hemoglobin A1c, use of statins, beta-blockers, angiotensin-converting enzyme inhibitors/angiotensin receptor blockers, metformin, or sodium-dependent glucose transporter 2 inhibitors. LRT, log-likelihood ratio test; MACEs, major adverse cardiovascular events.
